# Review of “Molecules that Changed the World” by KC Nikolaou and T Montagnon

**DOI:** 10.1186/1479-7364-6-16

**Published:** 2012-09-01

**Authors:** Panagiotis A Tsonis

**Affiliations:** 1 Department of Biology, University of Dayton, Dayton, OH, 45469-2320, USA

## Book details 

Nikolaou, KC and Montagnon, T

Molecules that Changed the World

Weinheim: Wiley-VCH; 2008

366 pages, ISBN 978-3527309832

Let me just say at the outset that I am a molecular biologist, and I have taught molecular biology for more than 20 years. My course requires organic chemistry. It seems that many students are at odds with organic chemistry and consider it difficult and demanding. Well, I will urge all of them to read this book. This is a beautiful book, extremely well thought, unique in style and easy to read. The book tells the story of the chemical synthesis of very important molecules in human health history, including aspirin, morphine, steroid and the pill cyclosporin, and Taxol®, which was one molecule that the authors synthesized chemically. For each molecule that they describe, the authors present a historical background, photographs of the main protagonists (we also need to know the faces and not only the names of the scientists who spend their lives for the benefit of humanity) and a description of the synthesis of the compound. I was fascinated to learn that salicin (the precursor of aspirin®) was advocated in ancient Egypt some 3,500 years ago. The ancient Egyptians knew about the beneficial effects of myrtle bark (which contains salicin) on rheumatism and back pain. Similar is the story for the discovery of steroids from Mexican yams. And if you are ready for some mystery and intrigue, read the story of strychnine and how it was used as a poison for murder, along with other alkaloids, such as hemlock, which was used to execute Socrates. The discovery of Taxol® was the result of a major effort in the USA from 1960 to 1981 to identify anticancer ingredients from natural sources (did you know that 110,000 substances from 35,000 plant species were tested?). However, natural compounds are often very difficult to isolate in large quantities from plants. Thus, Taxol's total chemical synthesis (led by the Nicolaou and Holton teams) provided a drug that was much needed. The book appropriately ends with chapters on small molecules and biologics (antiviral agents and vaccines) and their importance in many different genetic diseases. This is the style for all the molecules in the book. You do not need to be a chemist to enjoy and follow the book. I highly recommend this elegant and unique book for every scientist and even for nonscientists with a desire to learn the effects of scientific endeavors in human history. It is a major achievement for the field and rightly so foreworded by two Nobel Prize winners, E.J. Corey and Ryoji Noyori.

## Competing interests

The author declares that he has no competing interests.

